# Effect of ventricular fibrillation on infarct size after myocardial infarction: a translational study

**DOI:** 10.1007/s00395-024-01091-9

**Published:** 2024-11-23

**Authors:** Neven Stevic, Alexandre Pinède, Nathan Mewton, Michel Ovize, Laurent Argaud, Sandrine Lecour, Clément Boiteux, Thomas Bochaton, Martin Cour

**Affiliations:** 1 Service de Médecine Intensive –Réanimation, Hospices Civils de Lyon, Hôpital Edouard Herriot, 5 Place d’Arsonval, 69437 Lyon Cedex 03, France; 2 Faculté de Médecine Lyon-Est, Université de Lyon, Université Claude Bernard Lyon 1, Lyon, France; 3 INSERM UMR 1060, CarMeN, Lyon, IRIS France; 4 Institut de Cardiologie Des Hospices Civils de Lyon, Unité de Soins Intensifs Cardiologiques, Hôpital Louis Pradel, Lyon, France; 5 Centre d’Investigation Clinique, CIC 1407, Hôpital Louis Pradel, Lyon, France; 6 Cape Heart Institute, University of Cape Town, Cape Town, South Africa; 7 Institut de Cardiologie Des Hospices Civils de Lyon, Service de Rythmologie, Bron, France

**Keywords:** Ventricular fibrillation, Cardiac arrest, Infarct size, Myocardial infarction, Ischemia–reperfusion injury

## Abstract

Ventricular fibrillation (VF)-induced cardiac arrest frequently complicates ST-segment elevation myocardial infarction (STEMI). Although larger infarct sizes (IS) correlate with a higher risk of VF, the influence of VF itself on IS has remained poorly investigated. To address this knowledge gap, we analyzed the effect of VF on IS in patients and two experimental models. From a prospective cohort, 30 STEMI patients with VF were matched 1:2 with STEMI patients without VF on the common determinants of IS. The primary endpoint was IS, assessed using the 48-h area under the curve (AUC) for troponin. We also compared IS in pigs with/without spontaneous VF during STEMI (*n* = 15/group), and in an isolated rat heart model of myocardial infarction with/without electrically induced VF (*n* = 7/group). After matching, the patient characteristics, including the area at risk (AR), were similar. IS was 33% lower in the VF group compared to the control group (troponin AUC 1.6 [0.5–3.3] 10^6^ arbitrary units vs. 2.4 [0.9–4.1] 10^6^ arbitrary units; *p* < 0.05), but infarct scar size (assessed using MRI and ECG) did not differ between the groups at 1 and 6 months. In both experimental models, IS, expressed as a percentage of AR, was lower (*p* < 0.05) in the VF group than in the control group. When common determinants of IS are comparable, VF occurring prior to myocardial infarction reperfusion appears to be associated with smaller IS. Nevertheless, this finding, observed under specific experimental conditions and in a highly selected group of patients, was not associated with reduced infarct scar size.

Registration (HIBISCUS-STEMI cohort): ClinicalTrials.gov NCT05794022.

## Introduction

ST-segment elevation myocardial infarction (STEMI) is a common pathology affecting more than 600,000 patients each year in Europe and the USA, with a 1-year mortality rate of up to 15% [14, 33, 36]. Early reperfusion by primary percutaneous coronary intervention (PPCI) is currently the standard of care for STEMI and the most efficacious therapy for minimizing the infarct size (IS), which is a major determinant of morbidity and mortality [14, 30]. However, in many patients, early coronary revascularization with PPCI is insufficient to prevent death or heart failure. This has prompted intense research to identify additional therapies, such as ischemic conditioning, to further limit myocardial -ischemia–reperfusion injury (IRI) [13]. Unfortunately, translation of these therapies into clinical practice has been disappointing [3, 6, 13]. Potential reasons for this include the inclusion of patients at low risk for poor outcomes who receive optimal reperfusion therapy [3, 6, 13]. As a result, researchers have started to reconsider which patients are more likely to benefit from additional strategies targeting IRI [3, 6, 11]. A population of interest could be patients with STEMI complicated by cardiac arrest (CA) [3, 6].

CA complicating acute myocardial infarction is a frequent event (> 5%) [17]. In the majority of cases, ventricular fibrillation (VF) is the heart rhythm responsible for CA in STEMI patients [15]. Even with appropriate and successful resuscitation, STEMI complicated by CA due to VF is associated with much poorer prognosis than STEMI without VF [17]. This may be attributed, in part, to the greater extent of myocardial IRI. Indeed, VF is more likely to occur in patients with large amounts of ischemic or infarcted myocardium [15, 17, 35]. However, this does not necessarily mean that VF *per se* increases the extent myocardial infarction. In fact, most major determinants of IS (e.g., age, sex, culprit coronary artery, and duration of ischemia) are also risk factors for VF and thus can bias conclusions in observational studies [4, 15, 17, 24, 26, 35]. Interestingly, experimental studies, which have the advantage of better control for these confounding factors, have reported contradictory specific effects of VF (i.e., reduction or worsening of myocardial IRI) [8, 12, 28]. Therefore, it is essential to gain a more comprehensive understanding of the impact of VF-induced CA per se on myocardial damage to explore potential cardioprotective strategies for this high-risk group in the future.

The objective of this study was to assess the impact of VF on IS. To do so, we compared IS in STEMI patients with and without VF before PPCI, considering the key factors that affect IS. Additionally, to mitigate potential biases in observational clinical studies and explore the causal relationship between VF and IS, we examined the effects of both spontaneous and electrically induced VF on myocardial injury in two experimental models of myocardial infarction.

## Methods

### Clinical study

#### Study design

We conducted a matched-cohort study to assess the association between VF-induced CA and IS in revascularized STEMI. This clinical study was approved by the local institutional ethics committee (Comité de Protection des Personnes Sud-Est III, approval IRB number 2015-067B) and registered at ClinicalTrials.gov (NCT05794022). All subjects or their relatives gave their informed consent and all procedures we carried out in this study were in accordance with the ethical standards of our institutional research committee and the 1964 Declaration of Helsinki and its later amendments.

### Study population

Patients were selected from a prospective cohort (HIBISCUS-STEMI) of 458 consecutive patients with STEMI treated with PPCI, recruited between 2016 and 2022 at the cardiac ICU of the University Hospital of Lyon, France. The HIBISCUS-STEMI cohort has been previously described [2]. Briefly, this is a comprehensive database that encompasses clinical, biological, and imaging data including echocardiography and cardiac magnetic resonance imaging, as well as a biological collection. The patients are followed up for 6 months after STEMI. Patients were included if the Thrombolysis In Myocardial Infarction (TIMI) score [5] was < 3 at the beginning of PPCI, if the TIMI score improved by at least 1 point after PPCI, if the duration from chest pain onset to PPCI was less than 12 h, and if there were no missing data regarding troponin I levels. We first identified consecutive patients from the cohort who had both STEMI and VF before PPCI (VF group). These patients were then randomly matched in a 1:2 ratio to those without VF from the same cohort (with similar inclusion criteria). Exact matching of cases was achieved using four predefined variables known to influence IS and/or area at risk (AR) of IRI and/or occurrence of VF [2, 4, 15–17, 24, 26, 35]: age (> or ≤ 59 years), sex, culprit coronary artery, and duration of myocardial ischemia (> or ≤ 3 h) defined as the time from chest pain onset to PPCI.

### Data collection

Baseline data on demographic characteristics, risk factors and medical history, and blood levels of creatinine were collected. Additional data regarding VF-induced CA and cardiopulmonary resuscitation were reported according to the Utstein style [23].

The culprit artery was identified using angiography, and the TIMI score was determined both before and after PPCI. AR was angiographically estimated before revascularization using a modified version of the Alberta Provincial Project for Outcome Assessment in Coronary Heart Disease (APPROACH) score [21], expressed as a percentage of the left ventricle (LV). Blood levels of cardiac troponin I (cTnI) were measured using a chemiluminescence method on an Access II Immunoassay system (Beckman-Coulter, Fullerton, CA, USA) at four time points: upon admission, and at 4, 24, and 48 h after PPCI.

One month after STEMI, cardiac magnetic resonance imaging (CMRI) and echocardiography were performed. Six months after STEMI, vital status was determined for both groups, as well as the Cerebral Performance Category (CPC) score for the VF group [23]. The CPC score is a 5-point scale ranging from 1 (good cerebral performance) to 5 (death) used to estimate brain injury after CA; CPC 1 or 2 defines a favorable neurological outcome. An ECG was recorded at this timepoint.

### Infarct size

The primary endpoint was myocardial IS, as assessed by the 48-h area under the curve (AUC) for cTnI release (expressed in arbitrary units). This endpoint was selected, because, owing to the design of the cohort, minimal missing data regarding troponin were anticipated. Moreover, CMRI (the gold standard) was not performed for research purpose during the acute phase of STEMI in our center.

### Infarct scar size

Myocardial infarct scar size was assessed by CMRI 1 month after STEMI, using the CMRSegTools segmentation plugin (CREATIS, Lyon, France) with the OsiriX software (Pixmeo, Geneva, Switzerland), as previously described [9]. Infarct scar size was expressed as a percentage of LV mass.

At 6 months after STEMI, to assess the medium-term effects of VF on infarct scar, and in the absence of a CMRI planned in the research protocol, we examined ECGs (collected during routine consultations) for this purpose. Infarct scar size was estimated by ECG using both the modified Selvester and DETERMINE scores [10, 19]. The modified Selvester score was calculated using 37 ECG data and was expressed as a percentage of LV (number of points multiplicated by 3%) [10]. The DETERMINE score was calculated by summing the number of leads with Q waves (× 2), fragmented QRS, and T-wave inversion and was expressed in points (higher score indicates larger infarct scar size) [19].

## Experimental studies

### Spontaneous VF in an STEMI model in pig

We retrospectively compared IS in 30 pigs with (*n* = 15) and without (n = 15) spontaneous VF during acute myocardial infarction, previously included in the control groups of preclinical studies performed at the INSERM U1060 laboratory in Lyon, France. All experiments were approved by the State Committee of Animal Affairs. Animals included in experiments from 2006 to 2014 with a similar protocol for myocardial infarction (conducted by the same operator) were considered if (1) VF did not occur exclusively prior to ischemia or at reperfusion, (2) no pharmacological intervention was used to treat arrhythmias, (3) no technical issues were reported, (4) sustained arrhythmias were documented, and (5) both AR and IS data were available. The selection of animals was conducted with blinding to IS. Pigs were included in the VF group if they experienced at least one episode of successfully defibrillated VF during coronary occlusion. They were compared to controls that did not experience VF during both ischemia and reperfusion.

As previously described, male farm pigs (weighing 30 ± 3 kg) were anesthetized with ketamine, pentobarbital, and fentanyl [20]. They were then tracheotomized and ventilated. A median sternotomy was performed, and the left anterior descending coronary artery (LAD) dissected proximal to the first diagonal branch. A silk wire was passed around the LAD and threaded through a small vinyl tube to form a snare used to achieve coronary artery. The pigs were subjected to 40 min of ischemia, followed by 120 min of reperfusion. Ischemia was confirmed by regional epicardial cyanosis and ST-segment elevation on ECG, whereas reperfusion resulted in the disappearance of both signs. Sustained arrhythmias were recorded during both ischemia and reperfusion.

In the case of VF lasting ≥ 30 s (VF group), a 200–300 J defibrillation was achieved using external paddles from a Sirecard-F defibrillator (Siemens, Erlangen, Germany). No antiarrhythmic drugs or epinephrine was administered. The number of VF episodes, defibrillations, time from the onset of coronary occlusion to the first VF, and the total duration of VF were recorded.

### Induced VF in an ex vivo rat model of acute myocardial infarction

The effect of VF on IS was retrospectively assessed in an isolated rat heart model of acute myocardial infarction with or without electrically induced VF (*n* = 7 per group). The experiments were performed at the Cape Heart Institute, Cape Town, South Africa. The research was originally designed to examine the potential of melatonin in reducing myocardial IRI following VF associated with myocardial infarction. However, hearts were only included in the two groups used to set up the model because of the unexpectedly small IS in the VF group, limiting the possibility of observing a beneficial effect of melatonin. The study protocol was approved by the Animal Ethics Committee of the University of Cape Town (Approval No. 016–023).

Fourteen male Wistar rats (250–300 g) were anesthetized with intraperitoneal sodium pentobarbitone (60 mg/kg). As previously described, their hearts were quickly removed and retrogradely perfused using the Langendorff technique at constant pressure (100 cmH_2_O) with oxygenated Krebs-–Henseleit buffer at 37 °C [1]. The buffer was equilibrated with a mixture of 95% oxygen and 5% carbon dioxide and had the following composition (mmol/L): NaCl 118, KCl 4.7, CaCl_2_ 1.2, KH_2_PO_4_ 1.2, MgSO_4_ 1.2, NaHCO_3_ 25.2, and glucose 11.0 with a pH of 7.40. After a 30-min stabilization period, the hearts were randomly assigned to either the control or VF group. Coronary flow (CF) and left-ventricular developed pressure (LVDP) were measured. Hearts exhibiting a baseline heart rate < 250 beats/min, or CF < 8 ml/min or LVDP < 80 mmHg were excluded from the experiment at this stage. In both groups, the left coronary artery was occluded with a 6/0 silk suture for 30 min followed by 120 min of reperfusion. In the VF group, VF was electrically induced at 5 min after coronary occlusion through electrodes placed on the heart cannula and left ventricle apex, using a pacemaker generator (PowerLab, ADInstruments, Sydney, Australia) set at 50 Hz, 8 V, 1 ms pulse duration; Krebs perfusion was also stopped to simulate CA. Ultrafast pacing was maintained for 30 s and stopped for 2 s to assess whether spontaneous VF was obtained (visually confirmed by disorganized ventricular wall motion with irregular LVDP fluctuations of very low amplitude). The same procedure was repeated in the absence of persistent VF. After 3 min of CA, Krebs perfusion was restored, and hearts were defibrillated if required by a flick of the forefinger to the right ventricle. A 3-min CA duration was selected to simulate a plausible clinical scenario, to reduce the risk of replicating ischemic conditioning, and ensure a high defibrillation success rate.

### Infarct size

In both animal models, the coronary artery was briefly re-occluded at the end of the reperfusion period, and a Uniperse blue dye was injected through the aorta. The hearts were then cut into 5–7 transverse slices, parallel to the atrioventricular groove. The non-ischemic myocardium appeared marked in blue, whereas the ischemic myocardium remained unstained [22]. Slices were then incubated in 1% triphenyltetrazolium chloride at 37 °C to differentiate the infarcted (pale) from viable (brick red) myocardial area [34]. IS and AR were quantified using computerized planimetry (ImageJ software). IS was expressed as a percentage of AR.

## Statistical analysis

Data are expressed as median with interquartile range [IQR] and number (percentage) except for sequential troponin measurements, which are shown graphically as geometric means. Categorical variables were compared using Fisher’s exact test, and continuous variables were compared using the t test or Mann–Whitney U test depending on the distribution of the data (evaluated using the Shapiro–Wilk test). Repeated measures were compared using two-way ANOVA and post-hoc Tukey’s test. The type II error (ß-error) was calculated for the cTnI AUC. Data were analyzed using GraphPad Prism 9 (GraphPad Software, La Jolla, CA, USA). Exact 1:2 matching on 4 binarized variables (age, sex, duration of ischemia, and culprit coronary artery) was performed using R V.4.3.3 software with the MatchIt package. Statistical significance was set at *p* < 0.05.

## Results

### Clinical study

#### Study population

In the HIBISCUS-STEMI cohort, 197/458 (43%) patients met the eligibility criteria, including 30 patients who experienced VF before PPCI. These 30 patients were matched with 60 patients without VF, and their baseline characteristics were similar (Table 1), except for creatinine levels which were significantly higher in the VF groups than in the control group (80 [65–99] vs. 68 [58–82] µmol/l; *p* = 0.03). The proportion of patients who received antiplatelet therapy between the onset of chest pain and PPCI was similar in both groups (Table 1). In the VF group, one (3%) patient received thrombolysis before PPCI but had a pre-PPCI TIMI flow of 0.

**Table 1:** Baseline characteristics of the matched cohort of patients with STEMI

	VF group (*n* = 30)	Control group (*n* = 60)	*p*
Age, years	59 [55–64]	61 [50–68]	0.77
Male	23 (77)	46 (77)	> 0.99
BMI, kg/m^2^	26 [25–30]	26 [23–30]	0.70
Hypertension	7 (23)	16 (27)	0.80
Diabetes	2 (7)	9 (15)	0.32
Hyperlipidemia	10 (33)	16 (27)	0.62
Current smoking	5 (17)	13 (22)	0.78
Previous myocardial infarction	3 (10)	4 (7)	0.68
Coronary angioplasty or CABG history	3 (10)	4 (7)	0.68
Stroke	0 (0)	2 (3)	0.55
Peripheral vascular disease	0 (0)	1 (2)	> 0.99
Usual treatment			
Aspirin	6 (20)	8 (13)	0.54
Beta-blockers	4 (13)	7 (12)	> 0.99
Anticoagulant therapy	0 (0)	0 (0)	> 0.99
Treatments started before PPCI ^a^			
Aspirin	20 (67)	37 (62)	0.82
Thienopyridine	15 (50)	35 (58)	0.50
Anticoagulant therapy	20 (67)	33 (55)	0.37
Culprit artery			
Left anterior descending artery	21 (70)	42 (70)	> 0.99
Right coronary artery	9 (30)	18 (30)	> 0.99
Pre-PPCI TIMI flow 0 or 1	24 (80)	50 (83)	0.77
Post-PPCI TIMI flow 2 or 3	29 (97)	60 (100)	0.33
Ischemic duration ^b^, min	157 [120–228]	166 [124–255]	0.51
Coronary angioplasty with stenting	29 (97)	55 (92)	0.66
Per or post-PPCI GP IIb/IIIa inhibitor ^c^	8 (27)	15 (25)	> 0.99

Data are expressed as median [first-to-third quartile] or number (%)

BMI: body mass index**;** PPCI**:** primary percutaneous coronary intervention**;** TIMI: thrombolysis in myocardial infarction**;** CABG**:** coronary artery bypass graft

^a^ Data were missing for 10 patients in VF group and 21 patients in control group, for each treatment

^b^ Time between chest pain onset and PPCI

^c^ GP IIb/IIIa inhibitors were Abciximab or Tirofiban

Characteristics of CA resulting from VF are shown in Table 2. Duration of VF was 2.5 (1.0–5.0) min. The seven patients (23%) who required invasive mechanical ventilation were all in a comatose state after return of spontaneous circulation (Table 2). Six patients (20%) required continuous administration of vasoactive drugs (dobutamine or norepinephrine) during the post-CA period.

**Table 2:** VF-induced cardiac arrest characteristics

	VF group (*n* = 30)
Number of cardiac arrests	1 [1–1]
Number of defibrillations	1 [1–3]
Place of cardiac arrest	
Place of residence	1 (3)
Public place	5 (17)
Pre-hospital medical care	15 (50)
In hospital	9 (30)
Witness	30 (100)
Untreated cardiac arrest, min	1 [1–1]
Time from start of CPR to ROSC, min	4 [1–15]
Epinephrine	3 (10)
Invasive mechanical ventilation	7 (23)

Data are expressed as median [first-to-third quartile] or number (%)

VF: ventricular fibrillation; CPR: cardiopulmonary resuscitation; ROSC: return of spontaneous circulation

### Infarct size

**Fig. 1:**
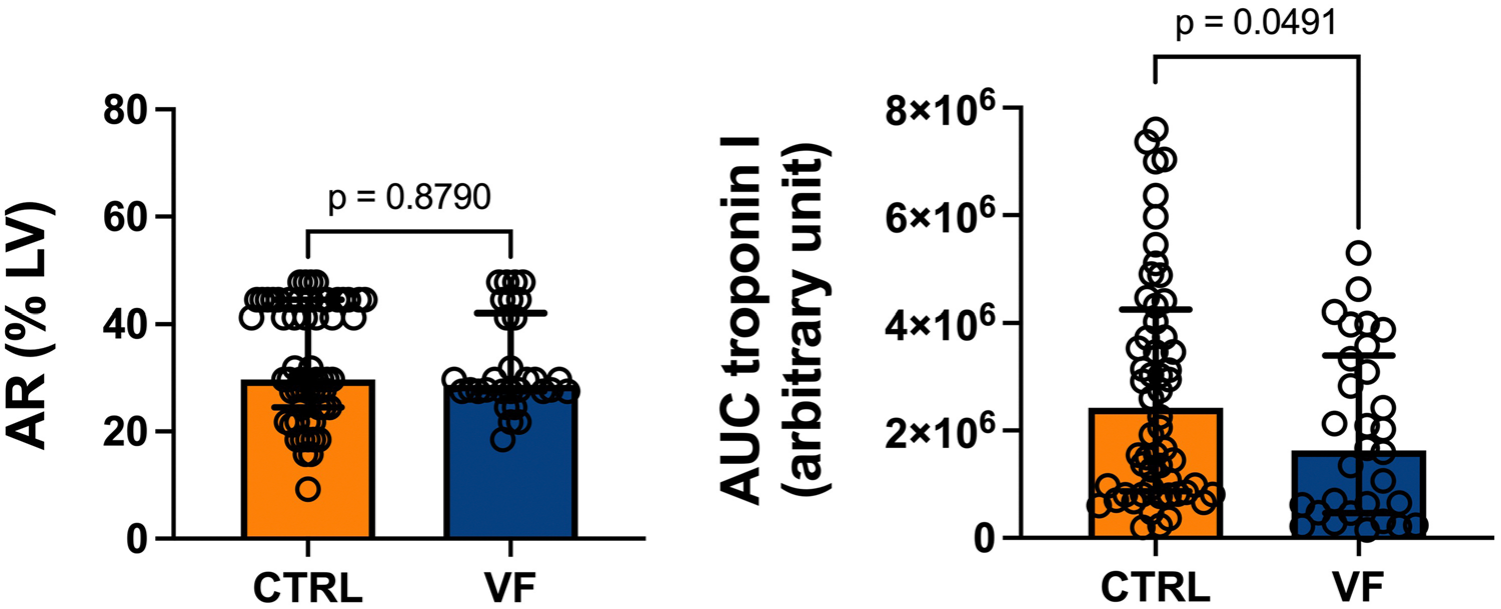
Effect of ventricular fibrillation on infarct size in patients with STEMI The area at risk (AR), expressed as a percentage of the left ventricle (LV), did not differ significantly between patients with STEMI and ventricular fibrillation (VF) and matched controls (CTRL) (left panel). The area under the curve (AUC) of 48-h troponin I was significantly lower in the VF group than in the CTRL group (right panel). Data are expressed as median (first-to-third quartiles). The circles represent individual data

**Fig. 2:**
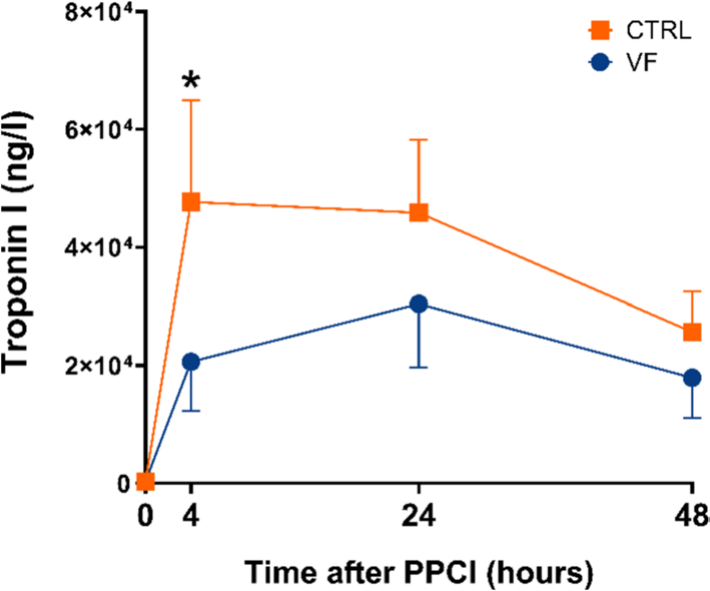
Effect of ventricular fibrillation on troponin kinetics in patients with STEMI Geometrical means and 95% confidence intervals of troponin levels are presented just before (0), and at 4, 24, and 48 h after primary percutaneous coronary intervention (PPCI) in the ventricular fibrillation (VF) group (blue dots) and the control (CTRL) group (orange squares). * *p* < 0.05 versus CTRL

As shown in Fig. 1, the AR were similar between groups (29% [28–41] in the VF group and 30% [25–45] in the control group; *p* = 0.88). The 48-h cTnI AUC was 33% lower (*p* = 0.049; ß-error = 0.37) in the VF group than in the control group (1.6 [0.5–3.3] 10^6^ vs. 2.4 [0.9–4.1] 10^6^ arbitrary units) (Fig. 1). The cTnI levels were also lower at 4, 24, and 48 h after STEMI in the VF group, although the difference was only significant at 4 h (Fig. 2). There was no correlation between VF duration and IS (R-square = 0.07; *p* = 0.15*).*

### Secondary outcomes

One month after STEMI, the CMRI measurements of infarct scar sizes did not significantly differ between the two groups (Table 3). Six months post-STEMI, the infarct scar sizes estimated by ECG were similar between the two groups (Table 3). At 6 months post-STEMI, all patients but one were alive, and all patients in the VF group had good neurological outcomes (Table 3).

**Table 3:** Secondary outcomes

	VF group (*n* = 30)	Control group (*n* = 60)	*p*
Troponin I peak, × 10^3^ ng/L	55 [13–115]	73 [30–138]	0.08
Echocardiographic LVEF at 1 month ^a^, %	56 [45–60]	55 [46–64]	0.55
CMRI at 1 month ^b^			
Infarct scar size, % of LVM	16 [6–29]	22 [12–32]	0.38
LVEF, %	50 [46–55]	50 [41–55]	0.66
LVEDV, mL	179 [142–211]	186 [158–203]	0.68
ECG estimated infarct scar size at 6 months^c^			
Selvester score, % of LVM	18 [9–27]	24 [12–30]	0.09
DETERMINE score, arbitrary unit	5 [2–7]	4 [2–7]	0.79
Alive at 6 months	30 (100)	59 (98)	> 0.99
CPC score 1–2 at 6 months ^d^	30 (100)	–	–

Data are expressed as median [first-to-third quartile] or number (%)

VF: ventricular fibrillation; LVEF: left-ventricular ejection fraction; CMRI: cardiac magnetic resonance imaging; LVM: left-ventricular mass; LVEDV: left-ventricular end diastolic volume; CPC: cerebral performance category

^a^ Data were missing for 1 patient in VF group and 4 patients in control group

^b^ Data were missing for 12 patients in VF group and 16 patients in control group

^c^ Data were missing for 4 patients in control group, 1 patient of VF group was excluded from analysis due to right bundle branch block with QRS ≥ 120 ms

^d^ Data collected only for VF group

#### Experimental studies

**Fig. 3:**
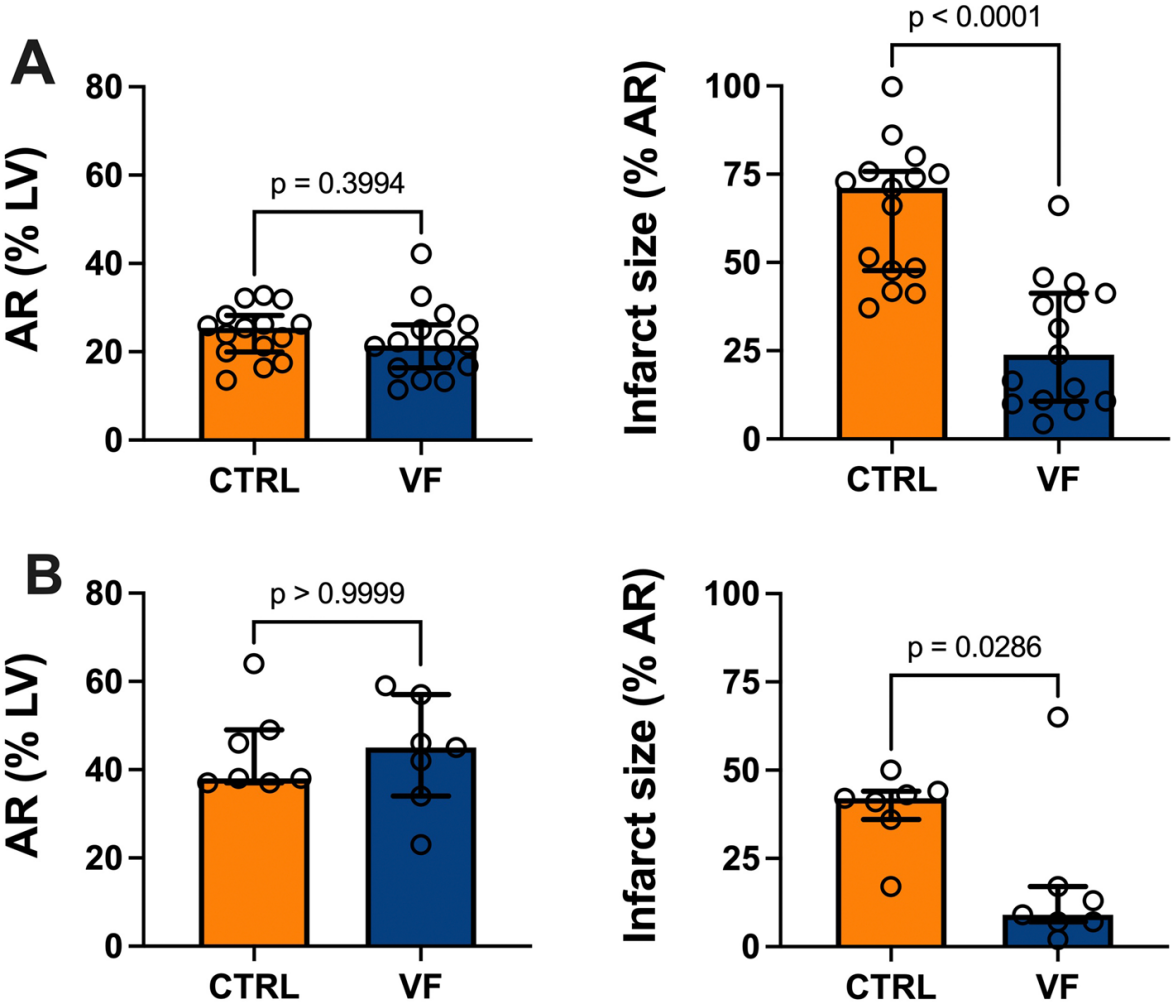
Effect of ventricular fibrillation on infarct size in animal models The area at risk (AR), expressed as a percentage of the left ventricle (LV), and the infarct size, expressed as a percentage of AR are presented for both the in vivo (A) and ex vivo (B) models of acute myocardial infarction with ventricular fibrillation (VF) or without VF (CTRL). Data are expressed as median (first-to-third quartiles). The circles represent individual data

In the pig model, the first episode of spontaneous VF occurred after 19 [15–23] min of ischemia. VF occurred 1 [1, 2] times and animals required 4 [2–8] electric shocks during ischemia. The total duration of VF per animal was 4 [1–6] min. In the control group, eight (53%) pigs presented ventricular extrasystoles and one ventricular tachycardia during coronary occlusion. During reperfusion, VF, ventricular tachycardia, and other sustained arrhythmias were observed in 3 (20%), 3 (20%), and 2 (13%) animals in the VF group and in 0 (0%), 10 (67%), and 4 (27%) animals in the control group, respectively. AR were similar between the VF and the control groups (Fig. 3A). IS/AR was 66% lower in the VF group than in the control group (24% [11–40] vs. 71% [48–75]; *p* < 0.001) (Fig. 3A). To assess the impact of time on these results, we compared the IS of the first eight animals included in each group with that of the next seven animals. IS was significantly lower in the VF group in both comparisons (*p* = 0.01 and *p* < 0.01, respectively).

In the isolated heart model, persistent VF following ultrafast pacing was obtained in 6/7 (86%) hearts. Of these, 5 required mechanical defibrillation. In the control group, during coronary occlusion, 1/7 (14%) hearts presented with spontaneous VF (requiring defibrillation) and 1/7 (14%) with ventricular tachycardia. CF and LVDP did not significantly differ between the VF and control groups at baseline (11 [10–12] vs. 11 [10–14] ml/min; 109 [84–116] vs. 118 [111–121] mmHg), upon coronary occlusion (7 [6–9] vs. 6 [6–10] ml/min; 71 [67–82] vs. 71 [62–75] mmHg), and at 120 min reperfusion (6 [6–9] vs. 6 [4–8] ml/min; 58 [41–71] vs. 60 [44–85] mmHg). AR was similar between the two experimental groups, while IS/AR was 79% lower in VF group compared to the control group (9% [7–15] vs. 42% [38–44]; *p* < 0.05) (Fig. 3B).

## Discussion

In this study, we found that STEMI patients experiencing VF prior to PPCI had significantly smaller IS than those without VF, after controlling for major factors that affect IS using exact matching. This finding was further supported by two experimental models of acute myocardial infarction, one involving spontaneous VF and the other involving electrically induced VF, which both reproduced the main outcome observed in the human cohort. Nevertheless, in the clinical study, the secondary outcomes (notably infarct scar sizes) were similar between the two groups.

Research comparing the extent of myocardial infarction in individuals with STEMI who experience VF prior to PPCI to those who did not is limited. Our study provides new and important data in this field by reporting both the 48-h AUC for cTnI release, a validated surrogate marker of IS, and direct measures of infarct scar size using CMRI, the gold standard in clinical practice. Importantly, the patients were carefully paired based on the key determinants of myocardial infarction size, which allowed the assessment of the effect of VF itself on IS. Considering these precautions, we report a reduction of approximately 30% in IS in patients with VF. Previous studies comparing STEMI patients with or without VF prior to PPCI only reported the peak levels of biomarkers of myocardial necrosis and did not consider confounding factors for IS. Ferrari et al. found that the creatine kinase (CK) peak was significantly higher in STEMI patients with VF than in STEMI controls, whereas CK-MB and troponin I peaks were not different [7]. Another study found that patients with STEMI and non-STEMI who experienced VF before PPCI had a higher peak of CK-MB in the control group, but there was no mention of other assessment of IS [15]. The primary shortcomings of these findings, which could account for the disparity with our results, include the fact that (1) VF patients and controls were not paired based on significant determinants of IS, (2) characteristics of patients were slightly different, (3) the CK and troponin peaks are less dependable than the AUC of troponin, and (4) CK may increase after CA because of a muscular damage caused by cardiopulmonary resuscitation. It is noteworthy that infarct scar sizes were similar between the two groups. This could be due to insufficient statistical power, as CMRI data were missing for 40% patients of the VF group. Alternatively, the decrease in IS may not have led to a smaller infarct scar size, which also depends on complex healing processes [13].

The characteristics of patients with CA in the present study were uncommon because of the selection criteria of the prospective HIBISCUS-STEMI cohort, which required informed consent. Indeed, all STEMI patients with VF had factors associated with a favorable prognosis, such as shockable rhythm, witnessed CA, bystander cardiopulmonary resuscitation, and were alive at 6 months. This contrasts with usual cohorts of patients with STEMI in which CA is associated with up to 40% mortality [17, 18, 27]. Moreover, most of the patients in our study were conscious after defibrillation and did not require invasive ventilation or vasoactive drugs. This selection bias undoubtedly limits the generalizability of our results to all STEMI patients with CA. Further research should investigate the effects of VF on IS across a wider range of patient groups, particularly those who have experienced CA for a longer duration. In contrast, this selection has helped to obtain groups with similar characteristics after the matching process, facilitating the evaluation of the effects of VF itself on myocardial damage. A previous study assessed the effect of VF on survival in STEMI patients in a cohort that was even more highly selected than ours [29]. Indeed, only patients who experienced out-of-hospital VF-induced CA without coma after defibrillation were included and compared to matched controls (by age, sex, and culprit artery). The authors found that conscious patients following VF had better 1-year survival than controls. Although the authors did not provide any data on myocardial damage, this finding is consistent with our results, suggesting a potential protective effect of VF. Nevertheless, the better outcome in the VF group could be attributed to the shorter time lapse between the onset of chest pain and PPCI, emphasizing the significance of confounding factors in this area of research. Despite carefully matching patients on key determinants of IS, including the time from chest pain onset to PPCI, and confirming similar AR post-matching, we cannot rule out the influence of unaccounted factors on the results. Therefore, we also examined the effects of VF on IS in experimental models with better control over confounding variables.

The clinical cohort results were validated through the reanalysis of two independent experimental models, which showed a significant decrease in IS with VF. Using a translational approach, we mitigated the limitations of observational clinical studies. Notably, the animals in both VF and control groups were comparable in terms of age, weight, sex, coronary occlusion, myocardial ischemia duration, and reperfusion duration. Additionally, IS and AR were directly assessed using reference methods, providing more reliable results than indirect measurements [34]. Our in vivo model had the advantage of appropriately simulating the clinical scenario of ischemia-induced VF and closed-chest defibrillation. However, a previous study found that pigs experiencing spontaneous VF had larger IS [28], implying that the experimental conditions may influence the outcomes. This difference in results may be due to the immediate defibrillation of animals in the previous study, whereas in our study, defibrillation was applied after a 30-s delay, potentially inducing a protective ischemic conditioning stimulus. Previous studies have suggested that VF induces conditioning [12, 25, 32]. Brief episodes of electrically induced VF before sustained ischemia (preconditioning) have been shown to prevent post-ischemic myocardial dysfunction in rats both in vivo and ex vivo [12, 32]. Another ex vivo study on myocardial infarction found that electrically induced VF was as effective as ischemic postconditioning in limiting IS [25]. Our research on isolated rat hearts extends these findings by demonstrating that per-ischemic VF reduces IS. This model also allowed precise control over VF onset and duration, reducing confounding factors and eliminating the need for defibrillation, thus isolating the effects of VF on IS. However, as coronary perfusion was stopped during VF, we cannot exclude that part of protection was related to ischemic conditioning rather than VF (or a combination of the two stimuli). Ischemic conditioning may have also participated to the protection observed in STEMI patients and in the pig model as VF was associated with brief cessation of circulation (whole-body ischemia) followed by reperfusion.

Our study has implications for clinical practice and translational research. A novel and counterintuitive finding is that VF-induced CA, at least when rapidly reversed, does not increase IS. This knowledge could assist clinicians in better understanding the cardiac short-term outcomes of such patients, notwithstanding the absence of significant differences in infarct scar size and the study's limitation in evaluating clinically relevant long-term outcomes (e.g., heart failure incidence, mortality). Another important finding is that VF might reduce IS in humans. This challenges the hypothesis that STEMI patients experiencing VF could be more suitable candidates than typical patients for studies on cardioprotective strategies targeting IRI [3, 6]. Additionally, the potential protective effect of VF should be considered when designing or interpreting the results of studies examining interventions aimed at decreasing IS. Importantly, these considerations may not apply to STEMI patients who present with a longer duration of CA. Although it is not conceivable to consider intentionally causing VF in patients as a treatment for STEMI, our findings open avenues for further investigation to elucidate the underlying protective mechanisms of VF. These mechanisms could potentially be replicated (e.g., pharmacologically) without inducing CA to prevent irreversible IRI in STEMI patients.

This study has several limitations. First, the sample size is relatively small and composed of highly selected patients, although this is the largest prospective study to report both a validated assessment of IS and an angiographic estimation of AR in STEMI patients with and without VF. Second, we used exact matching rather than propensity scores to pair patients, which may have drawbacks [31]. Exact matching pairs patients based on predefined critical variables, but increasing the number of matching variables to enhance precision, it may reduce the sample size and variability by excluding unmatched patients [31]. Nevertheless, in the present study, the method effectively created comparable groups without excluding any VF patients. Third, in the pig model, we did not measure myocardial blood flow and could not exclude the influence of this parameter on the IS. Finally, the potential mechanisms behind the protective effects of VF could not be explored due to the retrospective nature of the analyses. Additionally, we could not distinguish the direct effects of VF on IS from potential ischemic conditioning induced by brief global ischemia episodes during CA.

## Conclusion

Our research finding from a cohort of STEMI patients, in addition to results from two separate experimental studies, suggests that VF occurring before coronary artery reperfusion, is associated with a reduction in IS when compared to control subjects without VF but with similar determinant of IS. Although this decrease in IS was observed in highly selected patients and did not correlate with improved short- and medium-term outcomes (including reduction in infarct scar size), our findings suggest that IRI could be mitigated in the human heart and offers new avenues for research in cardioprotection.

## Data Availability

The data that support the findings of this study are available from the corresponding author, MC, upon reasonable request.

## Abbreviations

AR: Area at risk
AUC: Area under the curve
CA: Cardiac arrest
CF: Coronary flow
CK: Creatine kinase
CMRI: Cardiac magnetic resonance imaging
CPC: Cerebral performance category
cTnI: Cardiac troponin I
IRI: Ischemia reperfusion injury
IS: Infarct size
LAD: Left anterior descending
LV: Left ventricle
LVDP: Left-ventricular developed pressure
PPCI: Primary percutaneous coronary intervention
STEMI: ST-segment elevation myocardial infarction
TIMI: Thrombolysis in myocardial infarction
VF: Ventricular fibrillation
